# Supplementary material for: Emerging Nanotechnology in Non-Surgical Periodontal Therapy in Animal Models: A Systematic Review
Source: Nanomaterials (Basel). 2020 Jul 20;10(7):1414. doi: 10.3390/nano10071414 (PMC7407288; doi:10.3390/nano10071414)
Supplement: Supplementary file 1 [file nanomaterials-10-01414-s001.pdf]

Supplementary Materials

TABLE

Table S1: Search equations.

Database	Search equation
Medline via PubMed, Web of Science, the Cochrane Library	(periodontium OR periodontology OR periodontitis OR "periodontal disease" OR "periodontal diseases" OR "periodontal therapy" OR “periodontal pocket”) AND (nanotechnology OR nanomedicine OR nanoparticle OR nanotechnologies OR nanoparticles)
ScienceDirect	(periodontology OR periodontitis OR "periodontal disease" OR "periodontal diseases" OR "periodontal therapy" OR “periodontal pocket”) AND (nanotechnologies OR nanoparticles OR nanomedicine)

Table S2: Reason for exclusion of studies.

**Excluded studies**	**Justification for exclusion**
Yao et *al.*, 2014<br>International Journal of Nanomedicine	Not reporting alveolar bone loss
Yao et *al.*, 2015<br>Journal of Colloid and Interface Science	Not reporting alveolar bone loss
Pramod *et al.*, 2015<br>Journal of Drug Targeting	Methodological issues
Botelho et *al.*, 2016<br>Phytotherapy Research	Not reporting alveolar bone loss
Alshammari *et al.*, 2017<br>Journal of Clinical Periodontology	Irrelevant studies
Bao *et al.*, 2018<br>ACS Nano	Methodological issues
Khajuria *et al.*, 2018<br>Archives of Oral Biology	Irrelevant studies
Ozdogan *et al.*, 2018<br>International Journal of Pharmaceutics	Irrelevant studies
Yadav *et al.*, 2018<br>International Journal of Biological Macromolecules	Irrelevant studies
Hu et *al.*, 2018<br>International Journal of Biological Macromolecules	Not reporting alveolar bone loss
